# Reviews

**Published:** 1884-03

**Authors:** 


### Reviews.

A COMPLETE HANDBOOK OF TREATMENT, Arranged as an Alphabetical Index of Diseases. Containing nearly 1,000 Formulæ. By WILLIAM AITKEN, M.D., F.R.S. 12mo, cloth, pp. 444. New York: Bermingham & Co. 1882. (Bermingham's Medical Library). $2.00.

The author of this little book is widely known through the medical world as the writer of the long celebrated "Aitken's Practice,"' and for those familiar with the latter work, the one in hand is at least superfluous. But, as all practitioners and most students cannot afford the leading treatises on practice, and as special works on pathology and diagnosis are readily accessible, the present volume of treatment is likely to become, as it well deserves, a most popular book with medical students.

The alphabetical arrangement of each article is a commendable feature which will be best appreciated by the busy practitioner who is in the habit of reading the treatment of each obsolete disease as a new case presents itself, and cares little for the pathology of the same. It is a well-known fact, that it is not so much a clear understanding of the case which establishes the reputation of the doctor, as a successful mode of treatment. Ten years ago, the mind of the profession was absorbed in the study of the pathological changes accompanying diseases to such an extent as to nearly forget that there was a remedy for each evil; but of late, the works of Bartholow and of Ringer in therapeutics, and the introduction of the antiseptics in surgery, have wellnigh made an entire revolution in the treatment of disease, and the sooner the practitioners of medicine adopt such methodical therapeutics as are laid down in this book, the better for their patients, and for hnmanity at large.

H. D. V.

A PRACTICAL TREATISE ON IMPOTENCE, STERILITY, AND ALLIED DISORDERS OF THE MALE SEXUAL ORGANS. By SAMUEL W. GROSS, A.M., M.D. Second edition, thoroughly revised, with 16 illustrations. 8vo., cloth, pp. 176.

This is an elegant monograph which has become celebrated as much for a peculiar idea of its author as for its intrinsic value. This idea is thus expressed in the preface to this second edition:

"As was stated in the first edition, of the affections discussed in this brochure at least two—impotence and spermatorrhœa—are commonly described as functional diseases of the testicles; while, according to my observations, they usually depend upon reflex disturbances of the genito-spinal center, and are almost invariably induced or maintained by appreciable lesions of the prostatic portion of the urethra, which, as they may not be perceived by the patient, are frequently overlooked by the physician."

This is the old theory of Lallemand revived and better supported, and so also the latter's treatment, applied with some improved methods, is highly recommended.

Unusual attention is appropriately given the subject of sterility in the male. In the chapter on Sterility, the abnormal conditions of the semen and the causes which deprive it of its fecundating properties are fully considered—a portion of the work intended to supplement the subject of sterility in the female. "From answers to letters addressed to many of the most prominent writers in this country on gynæcology, I find that, with few exceptions, the woman alone commands attention in unfruitful marriages. The importance of examining the husband before subjecting the wife to operation will be best appreciated when I state that he is, as a rule, at fault in at least one instance in every six."

We have read this practical and fascinating little book with unusual interest, and we found it full of valuable hints, although its perusal did not impart a conviction of the frequency of prostatic lesions, or of the prominence of masturbation as a cause of stricture, and it is possible that the views of the author may lead to unduly severe treatment in the hands of the quacks who monopolize in the large cities the specialty of venereal diseases.

At the same time we are well aware of, and desire to enforce the statement, that this is a most valuable monograph, which every practitioner ought to take cognisance of, for it throws much light on a subject as important as it is neglected.

H. D. V.

HANDBOOK OF THE DIAGNOSIS AND TREATMENT OF DISEASES OF THE THROAT, NOSE, AND NASO-PHARYNX. By CARL SEILER, M.D. Second edition. Thoroughly revised and greatly enlarged, with 77 illustrations. 12mo., cloth, pp. 295. Philadelphia: Henry C. Lea's Son & Co., 1883.

This is a little volume of rare merit, and few superfluous words will be found in its interesting pages. The well-earned reputation of Seiler, and his numerous communications to the medical press, render an extensive critique of his work useless, except in so far as it is advisable to state the various articles which it contains. The first chapter treats of the laryngoscope; the second, of the art of laryngoscopy; the third gives the anatomy of the parts; the fourth, the various instruments used besides the laryngoscope. Then comes catching cold, acute laryngitis, functional disorders of the larynx, neoplasms, pharyngitis, hypertrophy of tonsils, coryza, nasal catarrh; a comparative diagnosis of the preceding affections, and a bibliography.

Although so concise, it seems that each chapter is complete, and conveys the proper idea of the disease and its best modes of treatment. One feature of this book, which belongs in common to all the works published by Lea, is the clearness of type and of illustrations, the fine quality of the paper, and the neatness of the work. Of course, this treatise is better adapted for students, and not so well for practitioners, as some of the larger works treating of the same subject. H. D. V.

A PRACTICAL MANUAL OF THE DISEASES OF CHILDREN, WITH A FORMULARY. By EDWARD ELLIS, M.D. Fourth edition; revised and enlarged. 8vo., cloth, pp. 218. $1. New York: Bermingham & Co., publishers, 1882.

The well-known work of Ellis on Children here appears in a cheap form of crowded pages, a result of free trade in foreign books. No doubt, many poor practitioners will profit by that, as it is really wonderful that such a valuable book could be presented to them for the price of a dollar, and every practitioner, as well as student, should place it on the shelf of the library, for future reference at least. Indeed, few of the larger treatises on the subject contain as much diversified matter as this, and hardly any work on practice ever was written so methodically, and with as much regard for the practical side of treatment. H. D. V.

---

THE HEALTH OF DR. WILLIARD PARKER.—Dr. Parker, as many of our readers are aware, has been the subject of a number of attacks of illness during the past year or more, which have given rise to solicitude. He was lately seized with an acute sickness, and again there has been occasion for anxiety. Some improvement is now reported, and we trust that his exceptionally vigorous constitution will lead to his speedy recovery.—*N. Y. Med. Jour.*

ON Feb. 1, 1884, the New York Post-Graduate Medical School, at which, since its foundation, 140 students have availed themselves of the vast amount of clinical material placed at their disposal, will take possession of more commodious accommodations located on E. Twentieth St. This change is the more fortunate, as the building to be occupied is of such proportions as to admit of the establishment of a hospital service.

WE can state to our happy surprise that our Southern medical brethren are making a rapid progress in medical journalism. In Mexico the *Revista Medico-quirurgica* shows in its first number that this journal will be conducted as a first class journal, and the new journal of Caracas, *El Ensano Medico* proves the medical progress of this State.

GENEROUS RECOGNITION.—It is said that the Emperor of Brazil has given Prof. Lacerda $20,000 for his discovery of permanganate of potassium, hypodermically injected, as an antidote for the bite of the cobra.

